# Intestinal Microbiota Remodeling Protects Mice from Western Diet-Induced Brain Inflammation and Cognitive Decline

**DOI:** 10.3390/cells11030504

**Published:** 2022-02-01

**Authors:** Prasant Kumar Jena, Tahereh Setayesh, Lili Sheng, Jacopo Di Lucente, Lee Way Jin, Yu-Jui Yvonne Wan

**Affiliations:** 1 Department of Medical Pathology and Laboratory Medicine, University of California, Davis, Sacramento, CA 95817, USA; prasantjenacps@gmail.com (P.K.J.); tsetayesh@ucdavis.edu (T.S.); fine919@163.com (L.S.); dilucente@ucdavis.edu (J.D.L.); lwjin@ucdavis.edu (L.W.J.); 2 Department of Pediatrics, Cedars Sinai Medical Center, Los Angeles, CA 90048, USA

**Keywords:** gut microbiota, bile acid receptor, neuroplasticity, brain inflammation, metabolomics, *Bifidobacterium infantis*

## Abstract

It has been shown that the Western diet (WD) induces systemic inflammation and cognitive decline. Moreover, probiotic supplementation and antibiotic treatment reduce diet-induced hepatic inflammation. The current study examines whether shaping the gut microbes by *Bifidobacterium infantis* (*B. infantis*) supplementation and antibiotic treatment reduce diet-induced brain inflammation and improve neuroplasticity. Furthermore, the significance of bile acid (BA) signaling in regulating brain inflammation was studied. Mice were fed a control diet (CD) or WD for seven months. *B. infantis* was supplemented to WD-fed mice to study brain inflammation, lipid, metabolomes, and neuroplasticity measured by long-term potentiation (LTP). Broad-spectrum coverage antibiotics and cholestyramine treatments were performed to study the impact of WD-associated gut microbes and BA in brain inflammation. Probiotic *B. infantis* supplementation inhibited diet-induced brain inflammation by reducing IL6, TNFα, and CD11b levels. *B. infantis* improved LTP and increased brain PSD95 and BDNF levels, which were reduced due to WD intake. Additionally, *B. infantis* reduced cecal cholesterol, brain ceramide and enhanced saturated fatty acids. Moreover, antibiotic treatment, as well as cholestyramine, diminished WD-induced brain inflammatory signaling. Our findings support the theory that intestinal microbiota remodeling by *B. infantis* reduces brain inflammation, activates BA receptor signaling, and improves neuroplasticity.

## 1. Introduction

Western Diet (WD) intake leads to systemic inflammation and cognitive dysfunction [1,2]. Additionally, in a bodyweight gain independent manner, short-term consumption of a high-fat diet leads to hippocampal-dependent spatial memory impairments in rats [3,4,5]. Moreover, obesity is associated with reduced hippocampal volume and impaired hippocampal function [6,7]. Furthermore, WD significantly alters commensal bacteria in the gastrointestinal (GI) tract and influences brain physiology and behavior [1,8]. Moreover, intestinal bacteria also regulate enteric nervous system (ENS) development, and the Toll-like receptors (TLR2 and TLR4), which are regulated by intestinal bacteria, maintain the function of ENS [9,10]. Therefore, the gut microbiota has a significant role in the digestive as well as neuronal systems [1]. Gut microbiota directly stimulates the production of interleukin 1 β (IL1β) and tumor necrosis factor α (TNFα), which impairs hippocampal-dependent memories in rodents [11,12]. Together, the interplay between the gut and brain is a critical target for manipulating brain health and neurodegenerative diseases. 

It has been shown that probiotics regulate not only digestion but also neuronal function. Probiotics enhance intestinal epithelial integrity, reduce the inflammatory response, protect against barrier disruption, as well as inhibit neuroinflammation and neurodegeneration [13,14]. Supplementation with *Enterococcus faecium* and *Lactobacillus rhamnosus* reduces TNFα in vitro and enhances antioxidant enzymes in young mouse brains [15]. In a clinical study, supplementation of Lactobacilli and Bifidobacteria for 12 weeks improved cognitive and metabolic statuses in Alzheimer’s disease (AD) patients [16]. Similarly, supplementation of a mixture of Lactobacillus and Bifidobacteria improved both GI function and mood during pregnancy [17,18].

*B. infantis* has intestinal and extraintestinal health benefits [2]. *B. infantis* has the ability to metabolize human milk oligosaccharides that are essential for newborns [19]. Our previous study revealed the benefits of *B. infantis* in reducing hepatic inflammation and preventing cancer-prone nonalcoholic steatohepatitis [20]. It is unknown whether *B. infantis* can prevent diet-induced brain inflammation and provide beneficial effects to prevent cognitive decline. 

Modulating gut microbes using antibiotics also affects neuronal function. It has been shown that broad-spectrum antibiotic treatment reduces amyloid-β (Aβ) plaque deposition, attenuates plaque-localized glial reactivity, and alters microglial morphology in an AD mouse model [21]. Thus, it would be interesting to study whether antibiotics, which are known to reduce diet-induced liver inflammation [2,22], can also prevent brain inflammation. 

Bile acids (BAs) are among the leading mediators explaining how diet via the gut microbiota affects health. As the gut microbes produce secondary BAs, dysregulated BA synthesis is always accompanied by dysbiosis [2,22]. BAs can cross the blood-brain barrier (BBB) and affect brain function [23,24]. Moreover, it has been shown that AD pathophysiology is associated with the dysregulated production of both primary and secondary BAs [25]. Altered BA profiles were found in AD patients [26,27]. Whether BA signaling is implicated in diet-induced neuroinflammation before AD development remains to be examined. 

The current study examined whether gut microbiota remodeling with probiotic supplementation and antibiotics could reduce diet-induced brain inflammation. Our data revealed for the first time that *B. infantis* could attenuate pathological phenotypes caused by WD consumption, including brain inflammation and reduced neuroplasticity. In addition, WD-induced brain inflammation is attenuated by antibiotic treatment and reducing BA pool size. Together, our data signifies the impact of both gut microbiota and BA on brain function. Probiotic supplementation can potentially be used to prevent diet-induced cognitive decline. 

## 2. Materials and Methods

### 2.1. Mice Maintenance and Treatment Regimens 

Specific pathogen-free C57BL/6 mice were housed in steel micro-isolator cages at 22 °C with a 12-h light/dark cycle. To study the dietary effect, mice were provided a control healthy diet (CD; 5.2% fat, 12% sucrose, and 0.01% cholesterol, *w*/*w*, TD. 140415) or a WD (21.2% fat, 34% sucrose, and 0.2% cholesterol, *w*/*w*; TD. 140414) (Envigo, Indianapolis, IN, USA) after weaning (3 weeks, more than 4 mice per group were used in each experiment). Mice were euthanized by 5% isoflurane, USP (#NDC 13985-046-60, VetOne, Boise, ID, USA) for 5 min.

For intervention using probiotics, after eight months of WD-feeding, mice were randomly assigned to two groups to receive either *B. infantis* (10^9^ CFU/mL, daily oral) or PBS (control) for two months. Mice were euthanized at the age of 10 months. 

For antibiotic treatment, 7-month-old WD-fed mice received without or with broad-spectrum coverage antibiotics (ABX) consisting of Ampicillin (1 gm/L), Metronidazole (1 gm/L), Vancomycin (0.5 gm/L), and Neomycin (1 gm/L) in drinking water for 3 months while mice continued to be fed by WD. 

To study the effect of BAs, 8-month-old WD-fed mice received without or with cholestyramine (2% in WD). Experiments were conducted following the National Institutes of Health Guidelines for the Care, and Use of Laboratory Animals under protocols approved by the Institutional Animal Care and Use Committee of the University of California, Davis (Sacramento, CA, USA).

### 2.2. Gene Expression Profiling 

The hippocampal RNA was reverse transcribed into cDNA. qRT-PCR was performed on an ABI 7900HT Fast real-time PCR system using Power SYBR Green PCR Master Mix (Applied Biosystems, Foster City, CA, USA). The mRNA levels were normalized to the level of Gapdh mRNA.

### 2.3. Western Blot Analysis

Brain protein (40 mg) was subjected to SDS-PAGE under reducing conditions following the transfer to polyvinylidene difluoride membranes. The membranes were incubated with 5% nonfat milk, followed by incubation using a specific antibody. The following primary antibodies (dilutions) were used: CD11b (1:1000; Bioss Antibodies, Woburn, MA, USA), phospho-ERK1/2 and total ERK1/2 (1:1000; Cell Signaling Technology, Danvers, MA, USA), IL6 (1:1000, Bioss Antibodies, Woburn, MA, USA), TNFα (1:1000, LSBio, Seattle, WA, USA), postsynaptic density-95 (PSD-95, 1:1000; Cell Signaling, Danvers, MA, USA), brain-derived neurotrophic factor (BDNF, 1:1000; Millipore Sigma, St. Louis, MO, USA), GPBAR1 (1:3000, LSBio, Seattle, WA, USA) and β-Actin (1:10,000; Millipore Sigma, St. Louis, MO, USA). Then, membranes were incubated with horseradish peroxidase-conjugated secondary antibodies. The signals were detected using Pierce Super Signal West Pico chemiluminescent substrates (Thermo Fisher Scientific, Rockford, IL, USA).

### 2.4. Biochemical Analysis

Brain homogenates were used to measure IL1β and TNFα protein by ELISA according to the manufacturer’s instructions (R&D biosystem, San Jose, CA, USA). 

### 2.5. Electrophysiological Recording for Measuring Long-Term Potentiation (LTP) 

Electrophysiological recordings were performed as previously described [28]. Fresh coronal hippocampal slices (300 µm) were submerged in ice-cold oxygenated artificial cerebrospinal fluid (ACSF). The hemi-slices were transferred to the recording chamber and perfused with standard ACSF at a constant flow rate of ~2 mL/min. Field excitatory postsynaptic potentials (fEPSPs) were obtained from the stratum radiatum of the CA1 region of the hippocampus after stimulation. Extracellular recording electrodes were prepared from borosilicate capillaries with an outer diameter of 1.5 µm (Sutter Instruments, Novato, CA, USA) and filled with 3 M NaCl. The baseline stimulation rate was 0.05 Hz. The fEPSPs were filtered at 2 kHz and digitized at 10 kHz with a Multiclamp 700 B amplifier (Molecular Devices, Sunnyvale, CA, USA). Data were collected and analyzed with pClamp software version 10.3 (Molecular Devices, Inc., Sunnyvale, CA, USA). Slope values of fEPSPs were considered for quantitation of the responses. After 10 min of stable baseline, the recording of fEPSPs was evoked every 20 s, LTP was elicited by high-frequency stimulation, consisting of 2 trains of 100 Hz (1 s) stimulation with the same intensity and pulse duration used in the sampling of baseline fEPSPs. 

### 2.6. Brain Lipidomics 

Snap frozen frontal cortex specimens (10 mg) were homogenized using 1 mL of degassed, −20 °C cold solvent mixture of acetonitrile: isopropanol: water (3:3:2, *v*/*v*/*v*). Samples were centrifuged for 30 min at 14,000× *g, and* the supernatant was used for lipid extraction using an improved butanol–methanol method [29]. An aliquot of homogenate (20 μL) was thawed and transferred to a disposable glass tube followed by extraction using an organic solvent (200 μL, 3:1, butanol-to-methanol volume ratio) and another 200 μL of organic solution (3:1, n-heptane-to-ethyl acetate volume ratio) vertexing for 60 s, and ultrasound (60 Hz, 200 W) for 10 min. Then, the solution was stratified by adding ammonium acetate (50 mM, 200 μL) into it, vertexing for 60 s, and centrifuging (6000 rpm) for 10 min at 4 °C. The upper organic layer was shifted to a new tube and desiccated under a vacuum. At last, the dried samples were reconstituted with acetonitrile/isopropanol/water (100 μL, 3:4:1, *v*/*v*/*v*) and treated by ultrasound (60 Hz, 200 W) for 5 min. The clear solutions were removed for LC-MS detection performed by the UC Davis West Coast Metabolomics Center. Quality control samples were obtained by pooling and blending equal aliquots of each sample were detected. 

### 2.7. Gut Microbiota Analysis Using 16S rRNA Gene Sequencing 

Based on published methods, cecum content DNA was used for 16S rRNA sequencing [2,30]. Variable region four of the 16S rRNA gene was amplified and sequenced. Sequence reads were analyzed by QIIME based platform and/or demultiplexed and classified with a custom python application dbcAmplicons (https://github.com/msettles/dbcAmplicons) to identify and assign reads by expected barcode and primer sequences [31]. The Ribosomal Database Project Bayesian classifier was performed to assign sequences to phylotypes [32]. Reads were assigned to the first Ribosomal Database Project taxonomic level with a bootstrap score ≥ 50.

### 2.8. Untargeted Metabolomics Profile

Untargeted, semi-quantitative metabolomics profiling was performed using cecal metabolites. Gas chromatography time of flight mass spectrometry was conducted at the UC Davis West Coast Metabolomics Center based on published methods [30,33,34]. BinBase database was used to process acquired spectra, filtered, and matched with the Fiehn mass spectral library of 1200 authentic metabolite spectra with retention index and mass spectrum information or against the NIST library. Chemical similarity enrichment analysis was performed by ChemRICH [35]. Pathway analyses were generated by the open source website MetaboAnalyst 4.0 (Montreal, QC, Canada) [36]. 

### 2.9. Bile Acid Quantification

Serum BA quantification was performed based on the published method [2,37]. BAs were detected using an ultrafast liquid chromatography system (Shimadzu, Kyoto, Japan) coupled to an API 4000 QTRAP mass spectrometer (AB Sciex, Redwood City, CA, USA) operated in the negative ionization mode. Chromatography was performed on a Kinetex C18 column (50 × 2.1 mm, 2.6 µm; Phenomenex, Torrance, CA, USA), maintained at 40 °C, preceded by a high-pressure column prefilter. The mobile phase consisted of a gradient of methanol delivered at a flow rate of 0.4 mL/min. 

### 2.10. Bioinformatics and Statistical Analysis

Alpha-diversity, which summarizes the diversity of microbial structure, was analyzed within a sample for several alpha-diversity metrics, including species richness (Observed), Shannon, Inverse Simpson, and Fisher metrics, using the “Phyloseq” R package [38]. Beta-diversity, which summarizes the diversity between samples performed by weighted Unifrac distance, accounts for the abundance of the operational taxonomic units. The Kruskal–Wallis test calculated the differences between groups in microbiota genus level.

Cecal metabolomics data were analyzed with MetaboAnalyst and ChemRICH [34,39]. Brain lipidomics data were analyzed with MetaboAnalyst and R program. Data are expressed as mean ± SD. All other comparisons were calculated by two-tailed Student’s *t*-test, one-way ANOVA, or two-way ANOVA, followed by Tukey’s test using GraphPad Prism 8 software. *p* values are adjusted for multiple comparisons using a false discovery rate. *p* < 0.05 was considered statistically significant. 

## 3. Results

### 3.1. B. infantis Reduces diet-Induced Brain Inflammation 

**Figure 1:**
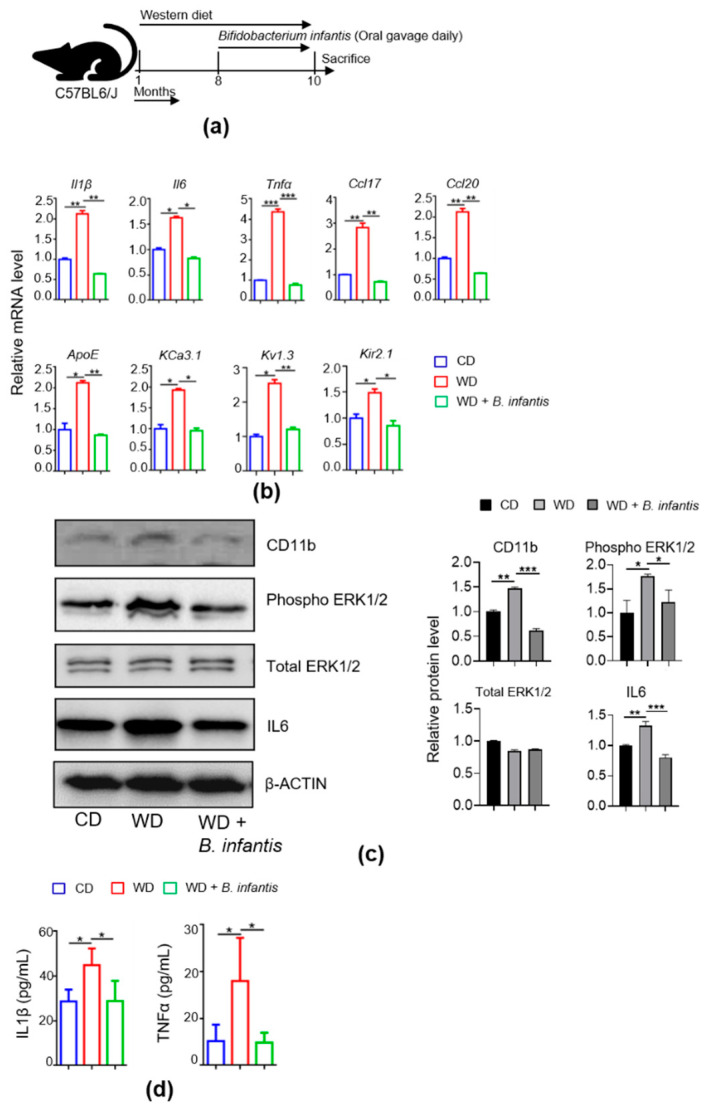
Probiotic *B. infantis* reduced brain inflammation signaling in WD-fed mice. (**a**) Experimental schema. (**b**) The hippocampal mRNA levels of inflammatory signaling genes of CD-fed (n = 6), WD-fed (n = 6), and WD-fed mice supplemented with *B. infantis* (n = 4). (**c**) Western blot of proteins related to inflammatory signaling. (**d**) Brain homogenate concentration of IL1β and TNFα by ELISA (n = 3). Data are expressed as means ± SD. One-Way ANOVA multiple comparisons Tukey *t*-test, * *p* < 0.05, ** *p* < 0.01, *** *p* < 0.001. CD-fed mice vs. WD-fed mice; WD-fed mice vs. *B. infantis* supplemented mice.

*B. infantis* supplementation for 2 months reduced WD-induced brain inflammation. The experimental scheme is shown in Figure 1a. WD-fed mice had increased *Il1β*, *Il6*, *Tnfα*, *Ccl17*, as well as *Ccl20*, and *B. infantis* reduced the expression levels. (Figure 1b). The mRNA level of *ApoE*, which is implicated in cholesterol homeostasis, increased in WD-fed mice, and *B. infantis* reduced it (Figure 1b). *B. infantis* reduced voltage-gated potassium channel mRNA levels of *KCa3.1*, *Kv1.3*, and *Kir2.1*, which were increased due to WD intake (Figure 1b). These ion channels play an essential role in brain inflammation and are a target in AD treatment. The protein levels of Phospho-ERK1/2, a potent effector of neuronal death and neuroinflammation [40], and CD11b, as well as IL6, were increased in WD-fed mice and decreased after *B. infantis* treatment (Figure 1c). In addition, ELISA data showed that brain IL1β and TNFα levels were increased in WD-fed mice, and *B. infantis* reduced the levels (Figure 1d).

### 3.2. B. infantis Improves Neuroplasticity in WD-Fed Mice

**Figure 2:**
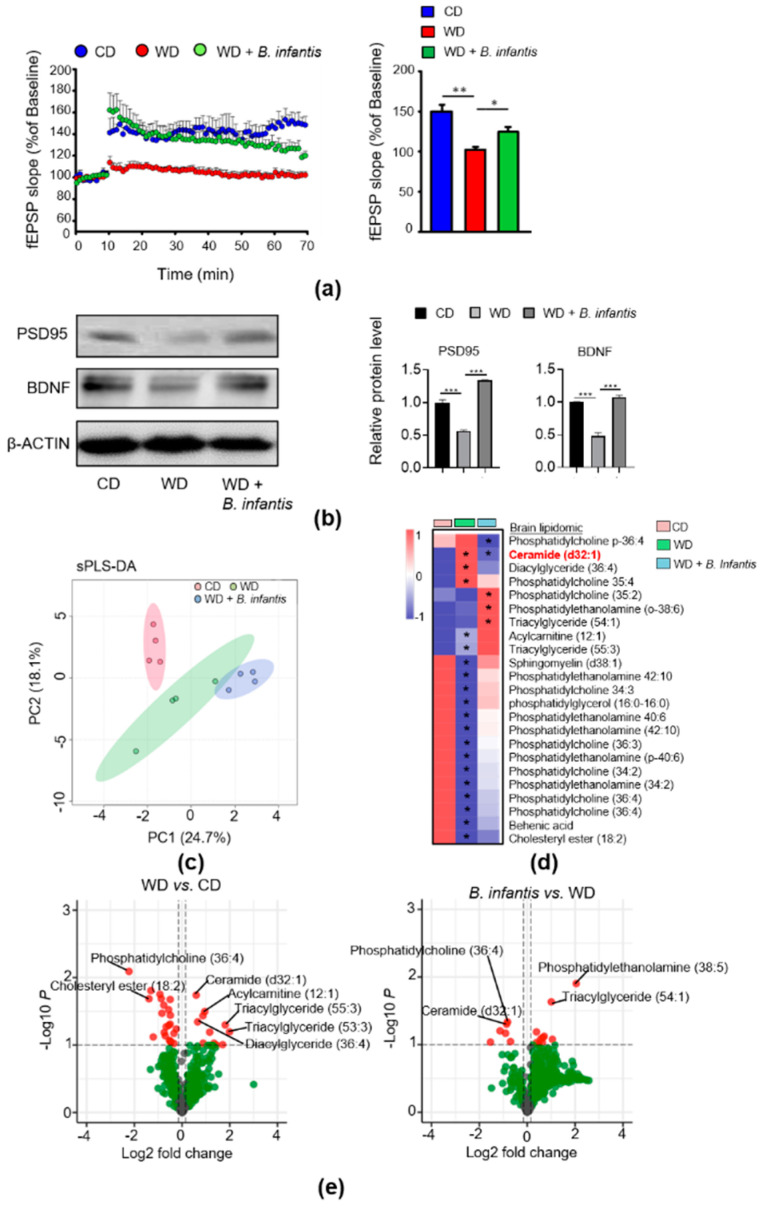
*B. infantis* treatment improved long-term potentiation (LTP) and synaptic deficits as well as an altered brain lipid profile: (**a**) Scatter plot indicates high-frequency stimulation-induced LTP, and the bar graph shows LTP calculated by averaging the change in fEPSP slope apparent between 50 and 60 min after high-frequency stimulation (n = 3, 9 slices per brain); all data is presented as the percent change in fEPSP slope means ± SEM from baseline; (**b**) Western blot data shows protein levels in the brains (CD, n = 6; WD, n = 6; WD + *B. infantis*, n = 4); (**c**) sPLS-DA based analysis of brain lipidomics; (**d**) Heatmap analysis shows a mean value of pick intensity of the top 23 lipids changed in each experimental group; (**e**) Volcano plots represent the brain lipidomics profile between WD vs. CD and WD + *B. infantis* vs. WD. The red color represents the fold changes of >2 with a *p*-value < 0.05, (n = 4). Data is mean ± SD. * *p* < 0.05, ** *p* < 0.01, *** *p* < 0.001.

We studied whether *B. infantis* supplementation could reverse reduced neuroplasticity based on LTP in WD-fed mice. After 2 months of *B. infantis* supplementation, LTP significantly improved compared to WD-fed mice. There was no significant difference in LTP between the control diet-fed healthy mice and *B. infantis*-supplemented WD-fed mice (Figure 2a). Moreover, WD intake reduced the levels of BDNF and PSD95, and *B. infantis* supplementation prevented those reductions (Figure 2b). 

### 3.3. B. infantis Supplementation Changes Brain Lipidomic Profiles in WD-Fed Mice 

In the brain, lipids are required for myelination and signal transduction [41]. A dysregulated lipid profile is associated with neurological disorders [42]. Brain lipidomic profiles were performed to study the effect of WD intake and *B. infantis* supplementation. Sparse partial least squares discriminant analysis (sPLS-DA) showed that brain lipid profiles formed three clusters based on the experimental groups (Figure 2c). 

Multiple unpaired *t*-tests revealed that ceramide (d32:1), diacylglycerol (36:4), phosphatidylcholine (35:4), acylcarnitine (12:1), and triacylglycerides (55:3) were increased in WD-fed mice (Figure 2d,e). WD intake also significantly reduced sphingomyelin (d38:1), phosphatidylethanolamine, phosphatidylcholine, phosphatidylglycerol (16:0-16:0), behenic acid, cholesteryl ester (18:2). *B. infantis* supplementation reduced phosphatidylcholine p-36:4 and ceramide (d32:1), however increased phosphatidylcholine (35:2), phosphatidylethanolamine (o-38:6), and triacylglyceride (54:1) (Figure 2d,e).

### 3.4. B. infantis Supplementation Modulates Cecal Metabolome

**Figure 3:**
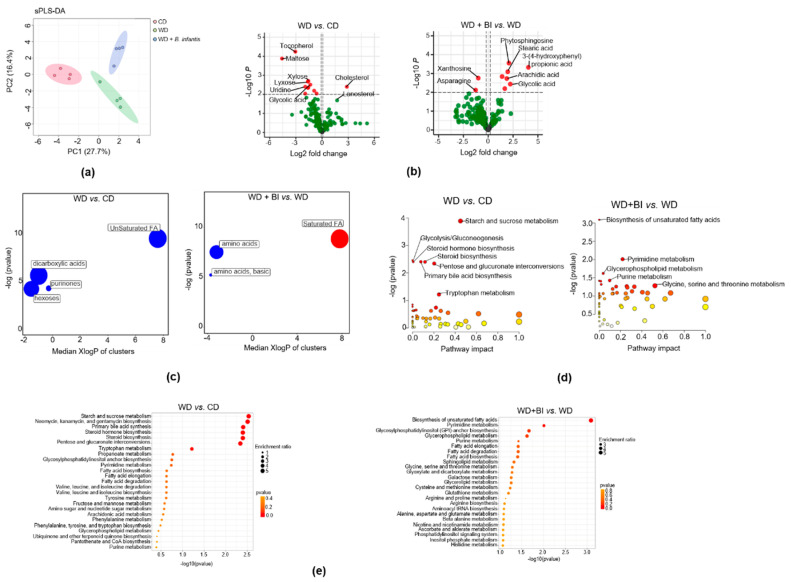
Untargeted metabolomics study of cecal content. Mice were fed a WD and treated with or without *B. infantis:* (**a**) sPLS-DA analysis of cecal metabolites clustered differently; (**b**) Volcano plots represent the cecal metabolomics profile between WD vs. CD (n = 4) and WD + *B. infantis vs*. WD (n = 4). The red color represents the fold changes of > 2 with a *p*-value < 0.05; (**c**) ChemRICH metabolite set enrichment statistics plot. The node color shows increased (red) or decreased (blue) metabolite sets. Only enrichment clusters are shown significantly different at *p* < 0.05 (The node sizes represent the total number of metabolites in each cluster set); (**d**) The pathway impact and (**e**) pathway analysis impacts were shown between WD vs. CD (n = 4) and WD + *B. infantis vs.* WD (n = 4).

GC-TOF-MS analysis of cecum metabolites revealed three distinct clusters based on experimental groups using sparse partial-least-squares discriminant analysis (sPLS-DA) (Figure 3a). Fold changes in the cecal metabolites between groups are shown in volcano plots (Figure 3b). 

WD intake increased cholesterol and lanosterol, and *B. infantis* treatment reduced them (Figure 3b). In addition, sugar substitutes such as xylose, maltose, and lyxose were reduced in WD-fed mice. Furthermore, *B. infantis*-treated mice had increased stearic acid (Figure 3b), a long-chain saturated fatty acid with neuroprotective effects [43]. 

Chemical similarity enrichment analysis showed that WD-fed mice had reduced unsaturated fatty acids, dicarboxylic acids, purinones, and hexoses (Figure 3c). The saturated fatty acid that rises in the brain during memory formation also increased in *B. infantis* treated mice [44] (Figure 3d,e). The top five relevant pathways between WD vs. CD groups were starch and sucrose metabolism, glycolysis/gluconeogenesis, steroid hormone/steroid biosynthesis, primary bile acid synthesis, and tryptophan metabolism based on MetaboAnalyst 4.0 (Figure 3d). On the other hand, biosynthesis of unsaturated fatty acids, pyrimidine metabolism, and glycine, serine, and threonine metabolism were among the top altered due to *B. infantis* supplementation (Figure 3d,e). 

### 3.5. B. infantis Supplementation Alters the Gut Microbiota of WD-Fed Mice 

**Figure 4:**
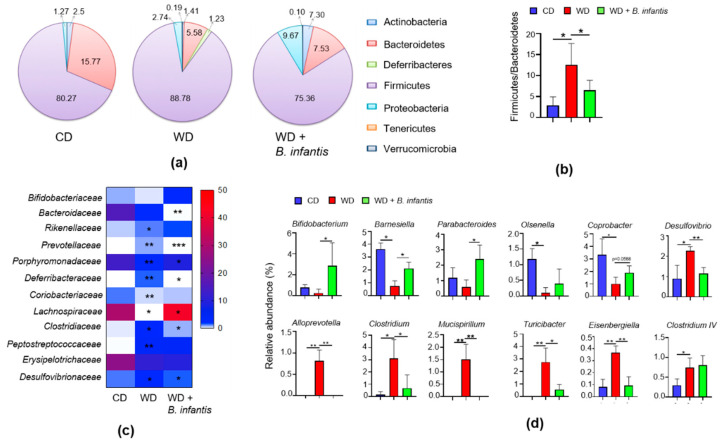
Probiotic *B. infantis* prevented gut dysbiosis induced by WD: (**a**) Relative abundance of cecal microbiota at the phylum level; (**b**) The ratio between phylum Firmicutes and Bacteroidetes; (**c**) Relative abundance of cecal microbiota at the family level; (**d**) Relative abundance of cecal microbiota at genus level in control diet (CD) and WD fed mice treated with and without *B. infantis* for 2 months (CD, n = 6; WD, n = 6; WD + B. infantis, n = 4). Data are expressed as mean ± SD. * *p* < 0.05, ** *p* < 0.01, *** *p* < 0.001, CD-fed mice compared with WD-fed mice and WD-fed mice compared with *B. infantis* treated mice.

We further studied the impact of WD and *B. infantis* supplementation on gut microbiota composition using 16S pyrosequencing. WD-fed mice had reduced Bacteroidetes, yet had increased Firmicutes leading to an elevated Firmicute to Bacteroidete ratio (Figure 4a,b). *B. infantis* supplementation reduced the ratio, mainly due to a reduction in Firmicutes. *B. infantis* also enriched Actinobacteria and Proteobacteria (Figure 4a). 

Under the Firmicutes phylum, WD-fed mice had increased Rikenellaceae, Clostridiaceae, and Peptostreptococcaceae, but reduced Lachnospiraceae (Figure 4c). However, *B. infantis* increased Lachnospiraceae, and reduced Clostridiaceae. Under the Bacteroidetes phylum, the abundance of Bacteroidaceae and Prevotellaceae was reduced in *B. infantis* treated mice. Under the Proteobacteria phylum, WD increased Desulfovibrionaceae, and *B. infantis* reduced it (Figure 4c). 

At the genus level, *B. infantis* significantly increased the abundance of Bifidobacterium, Barnesiella, and Parabacteroides (Figure 4d). WD-fed mice had increased Desulfovibrio, Alloprevotella, Clostridium, Mucispirillum, Turicibacter, Eisenbergiella, and *B. infantis* reversed those changes (Figure 4d). 

### 3.6. Microbiome Depletion by Antibiotics Reduce Brain Inflammation and Increase BA Receptor Signaling

**Figure 5:**
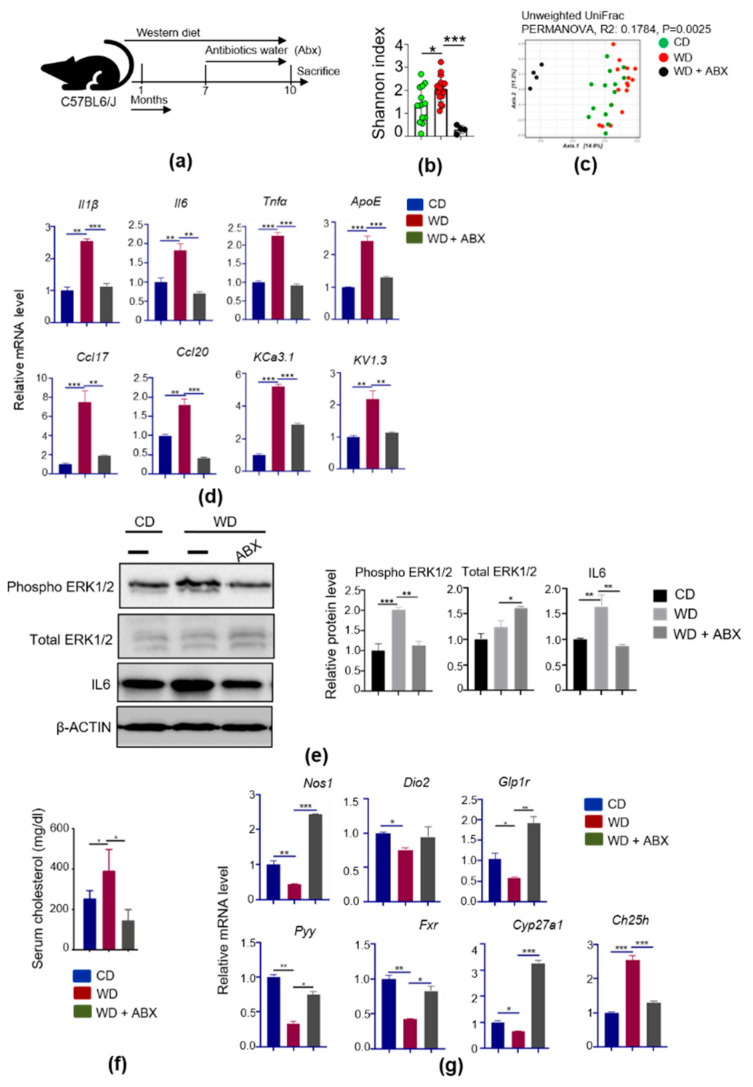
Microbiota depletion by antibiotics abrogated the inflammation induced by WD: (**a**) Experimental schema; (**b**) Shannon diversity index; (**c**) PCoA plot shows unweighted unifrac distance of fecal microbiota after 16S sequencing; (**d**) Relative mRNA level of inflammatory signaling genes in brain (CD, n = 6; WD, n = 6; WD with ABX, n = 4); (**e**) Western blot of inflammation signaling-related proteins in the brain of all experimental groups; (**f**) Serum cholesterol level and relative mRNA level of bile acid signaling genes in the brain of all experimental groups; (**g**) Relative mRNA level of bile acid receptor signaling genes in the brain. Data are expressed as mean ± SD. * *p* < 0.05, ** *p* < 0.01, *** *p* < 0.001, CD-fed compared with WD-fed mice, WD-fed mice compared with ABX (cocktail of ampicillin, neomycin, vancomycin, and metronidazole) treated mice.

We further studied the impact of antibiotics in influencing brain inflammation. The Shannon index (*p* < 0.05, Wilcoxon rank-sum test) was substantially reduced by ABX treatment (Figure 5a,b). A PCA plot of unweighted unifrac distance shows a distinctly diverse microbiome in the ABX group compared to untreated mice (PERMANOVA, R^2^ = 0.1784, *p* = 0.0025) (Figure 5c). Weighted unifrac is a quantitative measure of β-diversity, which clustered marginally differently between CD and WD fed mice (PERMANOVA, R^2^ = 0.16806, *p* = 0.001) (Figure S1a). ABX supplementation to WD-fed mice depleted fecal microbiota composition (Figure S1b,c). 

Real-time PCR data revealed that WD-induced inflammatory singling could be reduced by antibiotic treatment as evidenced by reduced *Il1β*, *Il6*, *Tnfα*, *ApoE*, *Ccl17*, *Ccl20*, as well as *KCa3.1* and *Kv1.3* in ABX-treated mouse brains (Figure 5d). Moreover, WD-activated ERK1/2 and IL6 induction was also reduced (Figure 5e), and serum cholesterol level was reduced by ABX treatment (Figure 5f). 

WD intake is expected to overburden BA signaling. Indeed, WD intake reduced the expression of hippocampal BA receptor GPBAR1 downstream genes, including *Nos1*, *Dio2*, *Glp1r*, and *Pyy*, as well as *Fxr*, and *Cyp27a1*, and microbiota depletion by ABX increased these BA receptors signaling genes (Figure 5g). Additionally, the expression of brain cholesterol 25-hydroxylase (*Ch25h*) was increased in WD-fed mouse brains, and ABX treatment reduced it (Figure 5g). These data unequivocally demonstrated the significance of the gut microbiota in regulating brain inflammation as well as BA signaling. 

### 3.7. B. infantis Supplementation Enhances BA Receptor Signaling

**Figure 6:**
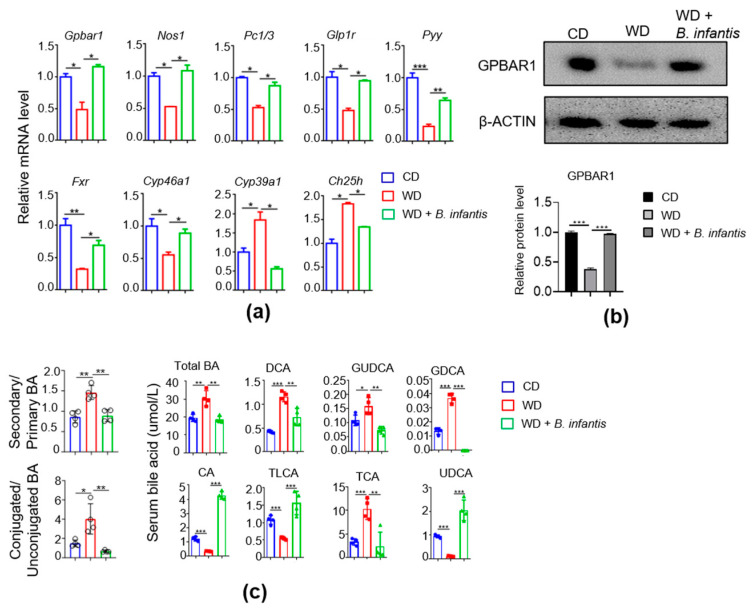
Probiotic *B. infantis* improved bile acid receptor signaling in the brain reduced by WD: (**a**) Bile acid signaling genes of CD (n = 6), WD (n = 6), and WD fed with *B. infantis* (n = 4) in mouse brains at the mRNA level; (**b**) The protein level of G-protein coupled bile acid receptor 1 (Gpbar1) in the brain; (**c**) Secondary to primary BA ratio, conjugated to unconjugated BA ratio, and serum bile acid level of CD, WD, and WD fed with *B. infantis* mice. Data are expressed as means ± SD. One-Way ANOVA multiple comparisons Tukey *t*-test, * *p* < 0.05, ** *p* < 0.01, *** *p* < 0.001. CD-fed mice compared with WD-fed mice and WD-fed mice compared with *B. infantis* treated mice.

We further studied the impact of *B. infantis* treatment on BA signaling in the brain. *B. infantis* supplementation increased brain *Gpba1*, *Nos1*, *Pc1/3*, *Glp1r*, and *Pyy* mRNA, which were reduced in WD-fed mice (Figure 6a). Additionally, brain *Fxr*, and *Cyp46a1* mRNA levels were increased, whereas *Cyp39a1* and *Ch25h* were reduced due to *B. infantis* supplementation (Figure 6b). Furthermore, WD intake reduced the protein level of GPBAR1, and *B. infantis* prohibited such a reduction (Figure 6c). 

Quantification of serum BAs revealed that WD-fed mice had an increase in total BAs, the ratio of conjugated-to-free BA, as well as secondary BAs, DCA, GUDCA, and GDCA, which were all reduced by *B. infantis* supplementation. Moreover, WD reduced CA, TLCA, and UDCA, which were increased by *B. infantis* (Figure 6c).

### 3.8. Bile Acid Sequestrant, Cholestyramine, Reduces WD-Indued Brain Inflammation

**Figure 7:**
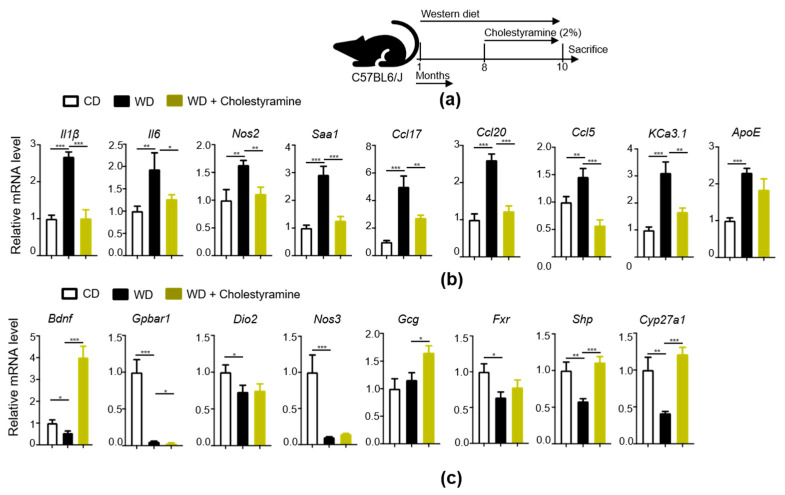
Effect of bile acid depletion by cholestyramine on brain inflammation and bile acid receptor signaling: (**a**) Experimental schema; (**b**) Relative mRNA level of inflammatory signaling genes in the brain (CD, n = 6; WD, n = 6; WD + cholestyramine n = 4); (**c**) Relative mRNA level of bile acid receptor signaling genes in the brain of all experimental groups. Data are expressed as mean ± SD. * *p* < 0.05, ** *p* < 0.01, *** *p* < 0.001, CD-fed compared with WD-fed mice, WD-fed mice compared with cholestyramine treated mice.

The experimental scheme is shown in Figure 7a. Reducing BA pool by cholestyramine could reduce hippocampal mRNA levels of *Il1β*, *Il6*, *Nos2*, *Saa1*, and chemokines *Ccl17*, *Ccl20*, and *Ccl5* as well as *KCa3.1* (Figure 7b). Moreover, cholestyramine treatment increased the *Bdnf* mRNA level compared to untreated mice (Figure 7b). Cholestyramine treatment also induced the expression of FXR target genes such as *Shp* and *Cyp27a1* (Figure 7c).

## 4. Discussion

This study revealed the benefits of probiotic *B. infantis* in preventing diet-induced cognitive decline in mice fed with a WD. Using antibiotic and cholestyramine treatments, the generated data also signifies the impact of gut microbiota and BAs in regulating brain inflammation.

Our data revealed that WD intake reduced LTP as well as the expression of BDNF and PSD-95. However, *B. infantis* supplementation reversed these effects. LTP measures learning ability and memory, and BDNF is necessary for memory persistence and storage [45]. Reduced expression of BDNF is implicated with the formation of neurotic plaques consisting of Aβ and neurofibrillary tangles [46]. Our data is in agreement with a previously published study that shows a reduction in BDNF in the hippocampus of HFD fed mice [47]. WD/HFD increases oxidative stress, which causes BDNF reduction [48,49]. *B. infantis* strain CCFM687 significantly improved behavioral test scores and increased BDNF level in the prefrontal cortex through the 5-HT1A-CREB-BDNF pathway [50]. PSD-95 protein determines the structural and functional integrity of excitatory synapses [51]. PSD-95 was also significantly downregulated in metabolically imbalanced neurons compared to the healthy condition [52]. 

Dyslipidemia considers a chronic risk factor for the progression of cognitive dysfunction [53]. In this study, we observed dyslipidemia in the brain of WD-fed mice, which was reversed by *B. infantis* supplementation. Lipidomic data showed that WD-fed mice had increased brain ceramide, and *B. infantis* treatment reduced it. Ceramides are lipid-soluble and readily cross the blood-brain barrier [54]. Ceramides are neurotoxic, and their levels are increased in AD [55]. Patients with more than one neuropathologic abnormality have higher levels of ceramides [56]. Obesity and insulin resistance produce neurotoxic ceramides and could account for cognitive impairment and neurodegeneration [54,57]. Additionally, reducing ceramide synthesis can protect mice from HFD-induced obesity and insulin resistance [58]. 

Microbiota depletion by broad-spectrum antibiotics improves dyslipidemia [59]. Microbiota depletion by ABX in APP/PS1 reduces brain Aβ deposition with an improved pattern of peripherally circulating cytokines, chemokines, and gut hormones [21]. In addition, healthy microbiota transplants reduce amyloid and tau pathology in AD mouse models [60]. Our data showed that ABX reduced brain inflammatory cytokines, suggesting gut microbiota play a role in brain dysfunction pathogenesis. Together, gut microbiome remodeling by probiotics and antibiotics prohibit reduced neuroplasticity caused by WD intake. 

This study showed WD-fed mice had high cholesterol levels and were minimized with *B. infantis* treatment. Cholesterol is associated with late-life cognitive function [61]. In the brain, cholesterol 24-hydroxylase (CYP46A1) controls cholesterol efflux and converts cholesterol to 24S-hydroxycholesterol, and 7α-hydroxylation is carried out by CYP39A1 [62]. The CYP46A1 gene may modulate the course of cognitive deterioration in later life [63]. CYP46A1 activity generates isoprenoids that are essential for LTP in the brain [64]. In consistency, *B. infantis* increased the level of CYP46A1 as well as LTP. Cholesterol clearance leads to BA synthesis, which helps in lipid absorption [65]. Primary BAs are derived from cholesterol, mainly in the liver, whereas secondary BAs are typically produced by bacteria in the gut [66]. It has been shown that increased secondary to primary BA ratio links AD development and cognitive decline [67]. 

Bile salt hydrolase (BSH) is a bacterial enzyme that catalyzes the hydrolysis of glycine- and/or taurine-conjugated bile salts into free unconjugated BAs [68]. *B. infantis* produces BSH. Quantitated PCR revealed that there are 24 copies of *Bsh* per ng of *B. infantis* DNA (our unpublished data). Thus, *B. infantis* supplementation increased unconjugated BAs and facilitated metabolism. In consistency, targeted metabolomic analysis of post-mortem brain samples identified higher ratios of glycochenodeoxycholate to CA as well as increased secondary BAs including DCA, LCA, TDCA, and GDCA in AD patients [25]. 

BA receptor FXR plays a critical role in regulating BA synthesis and regulating glucose, lipid, and energy homeostasis, influencing signaling pathways in the brain [69]. In addition, BAs also modulate GABAergic and N-methyl-D-aspartate receptor-mediated neurotransmission [70]. Our previous data showed that fructose-enriched WD induced gut and brain inflammation [2,34]. Moreover, reducing BA pool size effectively reduces skin inflammation and liver damage [71,72,73]. The data presented here indicate that WD-associated BAs are also implicated in neuroinflammation. 

In summary, our data reveal that the gut microbiome and BA signaling pathways play a significant role in the cognitive impairment of WD-fed mice. Gut microbiota remodeling with probiotics improves BA signaling, reduces inflammation, and boosts free fatty acids in the brain, which may provide a novel perspective on cognitive impairment induced by WD intake.

## Data Availability

The data presented in this study are available on request from the corresponding author.

## Supplementary Materials

The following are available online at https://www.mdpi.com/article/10.3390/cells11030504/s1, Figure S1. Microbiota depletion by antibiotic altered fecal microbiota profile.
