# Supplementary material for: Intestinal Microbiota Remodeling Protects Mice from Western Diet-Induced Brain Inflammation and Cognitive Decline
Source: Cells. 2022 Feb 1;11(3):504. doi: 10.3390/cells11030504 (PMC8834507; doi:10.3390/cells11030504)
Supplement: Supplementary file 1 [file cells-11-00504-s001.zip › cells-1566158-supplementary.pptx]

## Slide 1
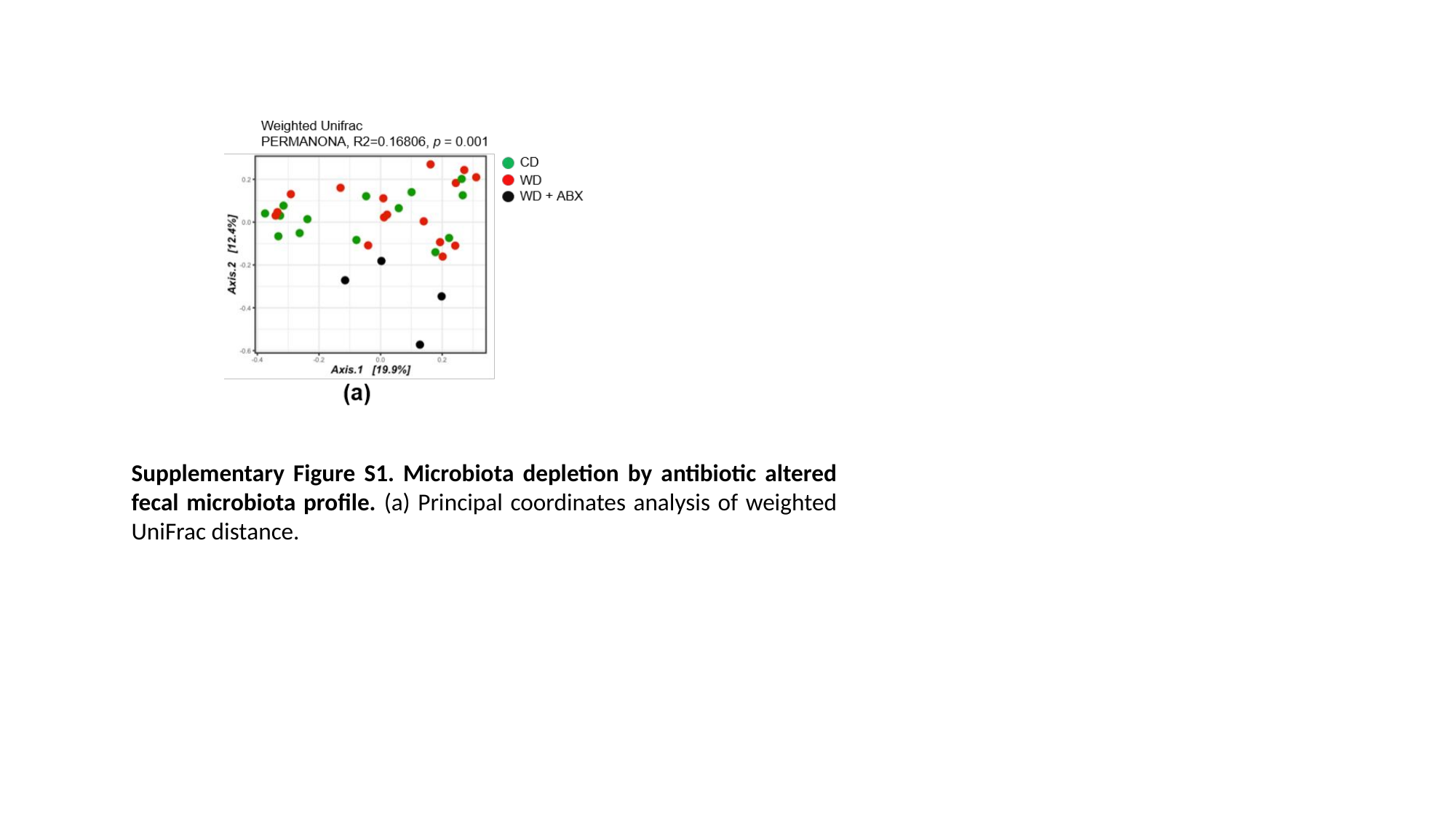

Supplementary Figure S1. Microbiota depletion by antibiotic altered fecal microbiota profile. (a) Principal coordinates analysis of weighted UniFrac distance.
